# Benefit of OTC Formula Against COVID-19 Is Explained by Selection Bias

**DOI:** 10.1177/2515690X211058417

**Published:** 2021-11-11

**Authors:** Harri Hemilä

**Affiliations:** 1 Department of Public Health, 3835 University of Helsinki, Helsinki, Finland

Margolin et al. published a study in which they concluded that a multi-component OTC formulation containing vitamin C, vitamin E, vitamin D, zinc, lysine, quercetin, and Quina extract prevented COVID-19.^1^ They reported that 9 of the 60 control participants became COVID-19 positive during the 20-weeks follow-up, whereas none of the 53 participants in the OTC regimen group became COVID-19 positive.

Participants were not divided into the groups randomly, nor by alternative allocation. Instead, participants of the “test” group decided for themselves to participate in the trial, whereas the “control” participants decided for themselves not to participate. Margolin argues that “subjects of the regimen-compliant test group and the non-compliant control group both met the same set of inclusion criteria”.^1^ However, “the same set of inclusion criteria” in this case does not make compliant and non-compliant participants similar. It is highly likely that there are systematic life-style and other differences between people who chose and those who do not chose to participate in intervention trials. In fact, there is empirical evidence for differences in people by willingness and compliance.

In the follow-up of the questionnaire-cohort of the Physicians’ Health Study, age-adjusted overall mortality was 19% lower among 59 277 men who were willing to participate, compared with 52 883 men who were not willing to participate in the trial.^2^ However, when baseline characteristics were taken into account, the adjusted difference fell to just 5% and was no longer a significant difference. Thus, essentially all of the significant 19% difference was explained by life-style and other differences between the two groups. For example, “those who were willing, tended to be younger, exercise more, and be less likely to have a positive disease history for several major chronic conditions”.^2^ Thus, the uniform set of criteria for sending the questionnaire to the large group of male physicians aged 40 to 84 years did not generate to a homogeneous group of men, and within the large group there were substantial systematic differences between those who were willing and those who were not willing to participate in the trial. In epidemiology this phenomenon is called selection bias. The purpose of randomization in randomized controlled trials (RCT) is to form two (or more) groups that do not have any systematic differences between the groups. Thereby the differences between the groups that appear during intervention can be attributed to the particular intervention.

There is RCT evidence that vitamin C may influence COVID-19,^3,4^ and that nasal carrageenan influences coronavirus infections.^5^ Therefore, randomized trials on OTC treatments for the new coronavirus and other respiratory viruses should be encouraged. However, comparison of participants who are willing versus not willing to participate in a trial is fundamentally biased and therefore the findings of the Margolin study are not a valid measure of the OTC regimen effect.^1^
